# ECAP‐Guided Closed‐Loop Spinal Cord Stimulation for Axial Thoracic Pain: A Case Report

**DOI:** 10.1002/ccr3.73785

**Published:** 2026-11-01

**Authors:** Aviraj Soin, Sarah Kassis, Richard Joseph Witte, Ben Sloop, Cameron Brittain

**Affiliations:** ^1^ The Ohio Pain Clinic Dayton Ohio USA; ^2^ Northeast Ohio Medical University Rootstown Ohio USA; ^3^ Wright State University Dayton Ohio USA; ^4^ Elite Pain Doctors Mason Ohio USA; ^5^ Ohio University College of Medicine Athens Ohio USA

**Keywords:** closed‐loop SCS, closed‐loop spinal cord stimulation, ECAP‐assisted closed‐loop SCS, thoracic pain, thoracic spine pain

## Abstract

Thoracic spine pain is among the least commonly reported pain syndromes. Conventional therapeutic strategies are frequently ineffective and may be associated with adverse events. Closed‐loop spinal cord stimulation (SCS) assisted with evoked compound action potentials (ECAPs) has emerged as a promising, safe and effective alternative. In this case report, we describe the successful use of ECAP‐controlled closed‐loop SCS in a patient with refractory thoracic spine pain. A female patient in her 40s presented with several axial thoracic pain refractory to all conservative and interventional therapies. Upon admission, the numeric rating scale (NRS) for pain score was 8/10. Functional limitations included restricted sleep duration (3 h) and limited standing and walking tolerance (20 min). The patient underwent a one‐week trial of ECAP‐controlled closed‐loop SCS, during which 24 million smart adjustments were automatically performed to maintain consistent neural activation. The dose accuracy was ±5 μV, with a target ECAP of 19.4 μV, and the measure was 16.4 μV, and the patient used the device 100% of the time. Following treatment, the patient reported improvement in pain and functional outcomes, with NRS scores decreasing to 2/10, and substantial improvements in sleep (6 h), standing, and walking duration. This case suggests that ECAP‐assisted closed‐loop SCS may provide meaningful pain relief and functional improvements in patients with refractory spine pain.

## Key Clinical Message

Thoracic spine pain is among the less prevalent pain syndromes. This case study describes the successful use of evoked compound action potentials (ECAP)‐controlled closed‐loop SCS in a patient with refractory thoracic spine pain. ECAP‐assisted closed‐loop SCS may provide meaningful pain relief and functional improvements in patients with refractory spine pain.

## 1 Introduction

Among spinal pains, thoracic pain is far less common than cervical or lumbosacral pain; however, it remains a clinically significant condition with a notable global health burden. Approximately 5% of patients referred to outpatient pain clinics present with thoracic pain as their chief complaint [1, 2, 3]. Thoracic pain commonly arises from occupational overload, sedentary behavior, poor posture, lack of regular physical activity, and degenerative joint pathology [2]. Studies have also identified associations with younger age, female sex, higher body mass index, elevated anxiety scores, cardiovascular disease, and prolonged sedentary postures such as reclining or sitting [4]. Despite its impact on quality of life and productivity, thoracic spine pain remains relatively understudied compared with other spinal pain syndromes [5].

Traditional management strategies for thoracic pain include pharmacologic therapy, physical therapy, and interventional procedures. Opioid analgesics are frequently prescribed but are limited by adverse effects, risk of diversion, and the potential for worsening pain over time, a phenomenon known as hypodopaminergic‐induced hyperalgesia, whereby pain sensitivity can be increased due to dopamine depletion [6]. These limitations have prompted interest in neuromodulation strategies as valuable tools in the management of thoracic spine pain, offering a safe, minimally invasive, and reversible alternative [7, 8].

Spinal cord stimulation (SCS) has demonstrated efficacy for the treatment of chronic thoracic spine pain [9]. Conventional SCS systems operate in an open‐loop manner, delivering fixed stimulation parameters that do not account for physiologic changes such as posture‐dependent shifts in the distance between electrodes and neural tissue [10]. These fluctuations can lead to inconsistent neural activation, resulting in episodes of under‐ or overstimulation and diminished therapeutic benefit [11].

Closed‐loop neuromodulation was developed to address these limitations by incorporating physiologic feedback into stimulation delivery. ECAPs represent measurable neural responses generated by dorsal column fibers resulting from stimulation pulses. It may also be used as a control signal in a closed‐loop SCS system for the adjustment of the parameters involved in the stimulation to reduce variability in the ECAP [10]. In ECAP‐controlled closed‐loop SCS systems, these signals are continuously monitored and used to automatically adjust stimulation intensity, thereby maintaining consistent neural recruitment despite physiologic variability [12, 13].

While ECAP‐controlled closed‐loop SCS has been explored in lumbar pain, its role in thoracic spine pain remains understudied. This case report describes the application of ECAP‐controlled closed‐loop SCS in a patient with refractory axial thoracic pain localized to the T6‐T9 dermatomal distribution.

## 2 Case History/ Examination

A female patient in her 40s, with a 32.1 kg/m^2^ Body mass index (BMI) presented with severe axial thoracic pain that was refractory to all prior treatment modalities. Previous interventions included physical therapy, pharmacologic management, spinal injections, nerve blocks, and ablations, which failed to provide meaningful pain relief. The pain was described as constant and debilitating, with an NRS score of 8/10 [14]. Functional impairment was substantial, with sleep limited to approximately 3 h per night and standing and walking tolerance restricted to 20 min.

## 3 Methods (Differential Diagnosis, Investigation and Treatment)

She was ultimately diagnosed with idiopathic neuropathic thoracic spine pain. The patient identified three primary therapy goals: (1) standing for longer than 20 min, (2) walking for longer than 20 min, and (3) sleeping for longer than 3 h per night.

Given the failure of previous treatments, the patient elected to undergo a spinal cord stimulator trial. A Saluda Medical closed‐loop SCS was implanted to maintain consistent neural engagement during these physical activities and prevent positional over‐stimulation. The lead (12‐electrode percutaneous lead) was placed targeting the superior articular facet of the T5 vertebrae, positioning the contacts 1–3 segments cephalad to pain distribution for optimal dermatomal coverage. Programming utilized a 12‐electrode configuration, which provided the necessary contact spacing for preferential activation at the top three contacts in a +/−/+ guarded cathode distribution to maximize field distribution. Stimulation parameters included a pulse width of 250 microseconds, a frequency of 40 Hz, and a maximum current of 13 mA. The patient noticed sensitivity at 40 μV/mA, and her ECAP target max was 60 microvolts. The ECAP target increment was 1 microvolt.

The patient completed a one‐week stimulation trial, during which approximately 24 million automated stimulation adjustments were performed. Dose accuracy remained within ±5 μV, and device utilization was recorded at 100% during the trial period (Figure 1).

**FIGURE 1:**
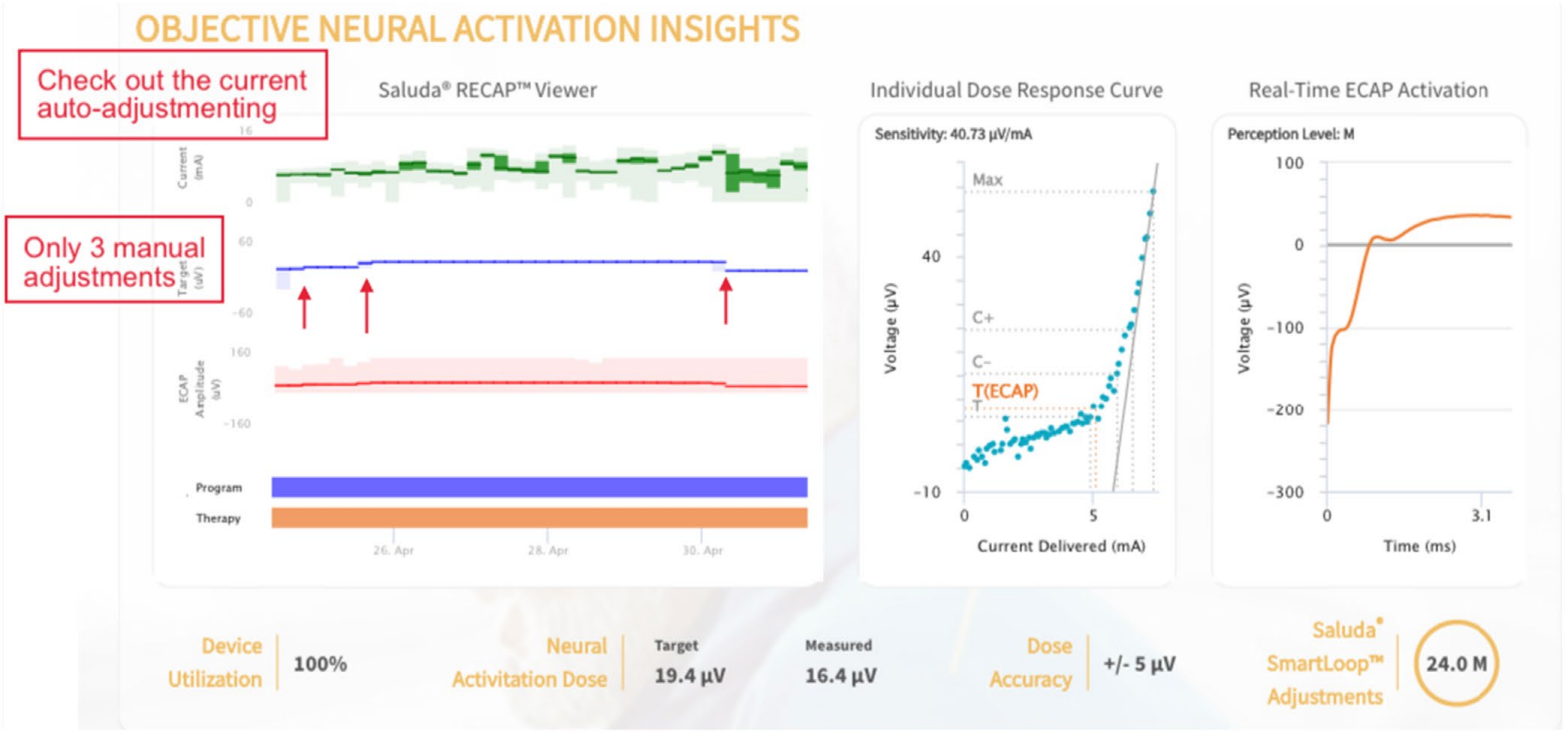
Closed‐loop stimulation performance demonstrated highly stable neural activation in a patient with thoracic spine pain.

## 4 Conclusion and Results (Outcome and Follow‐Up)

Following treatment, the patient achieved all predefined therapy goals. Sleep duration improved from 3 to 6 h per night, while standing and walking tolerance increased from 20 min to 6 h. Pain intensity improved substantially, with NRS scores decreasing from 8/10 to 2/10 (Figure 2). The patient underwent permanent implant with the lead placed at T5 under fluoro guidance (Figure 3).

**FIGURE 2:**
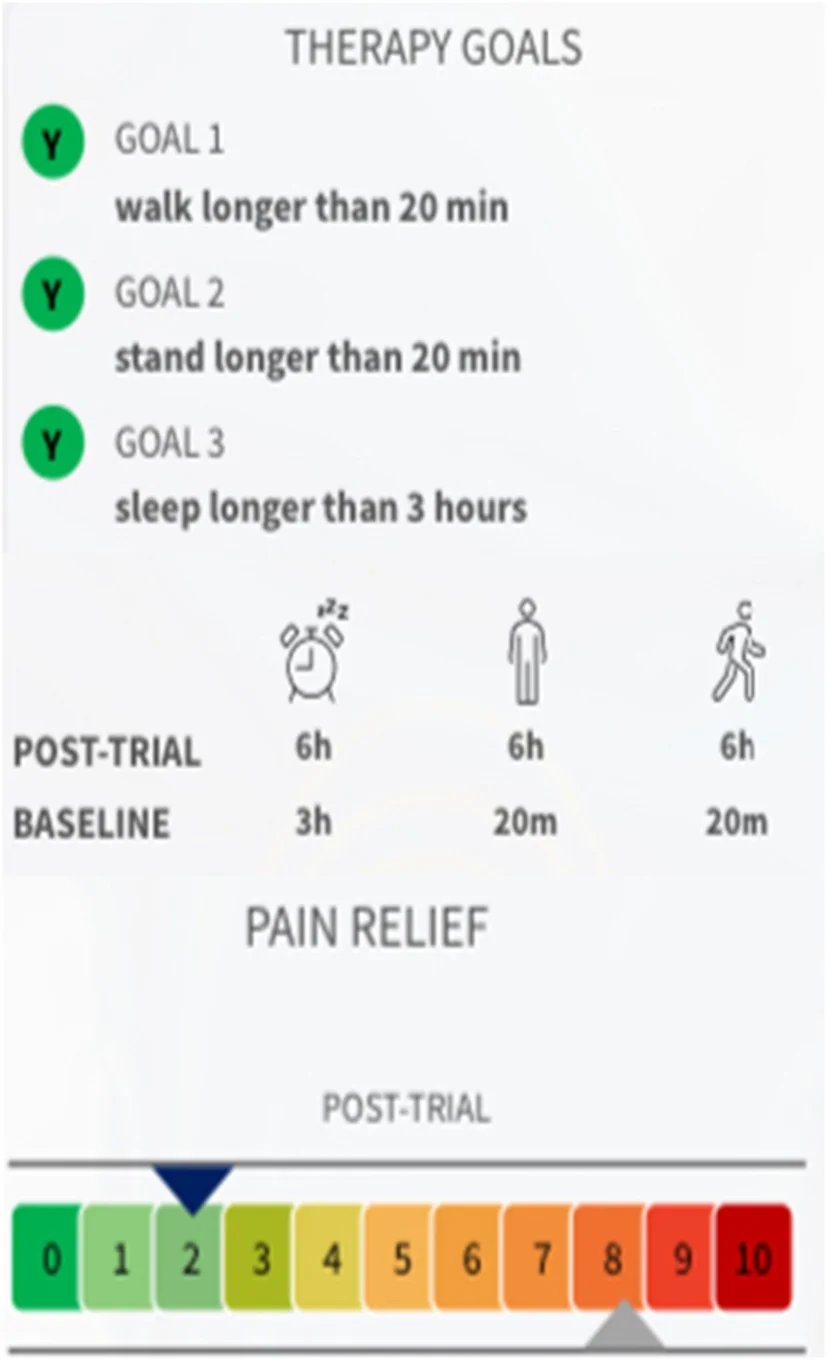
Outcomes after successful treatment with ECAP closed‐loop SCS.

**FIGURE 3:**
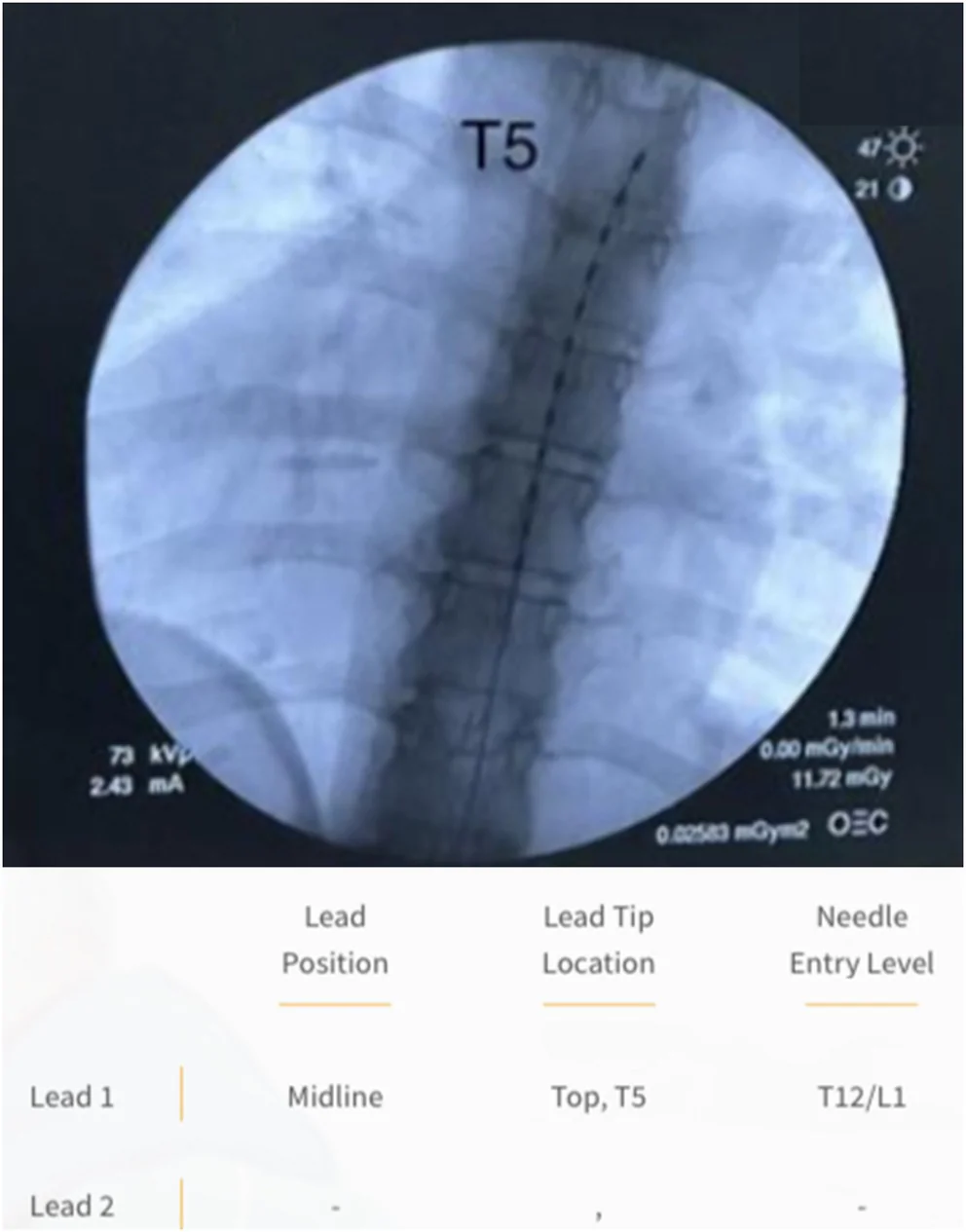
Fluoroscopic imaging of the spinal cord stimulator demonstrated lead spanning the T5 vertebral body.

## 5 Discussion

This case study highlights the potential role of ECAP‐based/controlled closed‐loop SCS as an effective and safe treatment approach for the management of refractory thoracic spine pain. While thoracic pain is less commonly targeted with SCS, the present case demonstrates meaningful pain reduction and functional improvement in a patient who had exhausted conventional treatment options. In the present case, a single 12‐contact lead was selected because the patient had localized pain to the midline axial thoracic region, and intraoperative testing demonstrated satisfactory dermatomal coverage, appropriate ECAP signals, normal impedances, and effective neural engagement with a single midline lead positioned at the T5 level.

Prior studies have reported favorable outcomes with thoracic SCS in select populations. Benedetti et al. described successful thoracic SCS use in pain following incomplete cervical spinal cord injury [15]. Larger observational studies of ECAP‐controlled closed‐loop SCS in chronic back pain have demonstrated sustained pain relief, at the 12‐month follow‐up mark, 81.4% of patients had ≥ 50% pain relief, while 53.5% had ≥ 80% pain relief with a high device utilization of 84.9% [16]. Similarly, another study reported significant pain reduction in patients treated with closed‐loop SCS for chronic pain conditions. After treatment, significant (*p* = 0.01) improvement was observed in the severity of pain (severity difference from baseline to the 3‐month follow‐up end: −18.95%, 95% CI, −3.1 to −0.5) [17]. The main difference between our case study and previously published studies is the focus on refractory thoracic spine pain, an indication that is less commonly treated with SCS.

Traditional therapeutic approaches, like physical therapy and psychotherapy, have been used for decades. Physiotherapists also indicated that for the effective management, a multimodal approach (education, manual therapy, therapeutic exercise) should be followed [18]. Meanwhile, the medical literature also supports such therapies as having a positive impact on patients with chronic pain, and these approaches are also widely recommended. However, these approaches can be used as an adjunct with SCS to provide quick pain relief to the patient [19].

The fundamental mechanism involved in the closed‐loop SCS ECAP‐guided neuromodulation is its ability to adjust the stimulation parameters to maintain and continuously measure dorsal column recruitment and dynamically adjust stimulation output in response to physiologic variability [20]. Unlike conventional open‐loop SCS, where current delivery or voltage is constant and dependent on the feedback of the patient, closed‐loop SCS adapts automatically to changes in posture, activity level, and cerebrospinal fluid dynamics that influence electrode‐neural distance [10, 21]. By maintaining consistent neural activation, ECAP‐controlled systems may reduce episodes of under‐ or overstimulation and improve therapeutic durability. ECAP‐controlled closed‐loop SCS resolves this problem by the measurement of neural activation directly, allowing the system to increase or decrease energy delivery for the maintenance of consistent therapeutic recruitment [22]. The pain relief in our patient may be an expected consequence of this physiologically responsive algorithm.

An additional benefit observed in this case was a reduction in programming burden. Closed‐loop SCS systems perform frequent automated adjustments, reducing the need for repeated clinician‐led reprogramming sessions and minimizing patient awareness of therapy variability. Open‐loop systems often require frequent parameter adjustments to compensate for postural changes, which may increase healthcare utilization and patient frustration [23]. The sustained pain relief observed in this patient without escalation of stimulation output further suggests that ECAP‐controlled closed‐loop SCS may mitigate tolerance or habituation, though this hypothesis warrants further study.

In conclusion, this case report demonstrates that ECAP‐controlled closed‐loop spinal cord stimulation may provide significant pain relief and functional improvement in patients with refractory thoracic spine pain. The adaptive, physiologically responsive nature of this therapy likely contributes to its clinical effectiveness by stabilizing neural recruitment despite physiologic variability. Larger prospective studies are warranted to further evaluate the role of ECAP‐controlled closed‐loop SCS in thoracic spine pain. Future studies with larger cohorts are needed to evaluate whether specific thoracic imaging characteristics can help stratify patients most likely to benefit from ECAP‐guided closed‐loop SCS for axial thoracic pain.

## Author Contributions

**Aviraj Soin:** conceptualization, data curation, formal analysis, investigation, methodology, project administration, resources, software, supervision, writing – original draft, writing – review and editing. **Sarah Kassis:** conceptualization, data curation, investigation, methodology, resources, visualization, writing – original draft. **Richard Joseph Witte:** conceptualization, formal analysis, investigation, validation, visualization, writing – original draft. **Ben Sloop:** conceptualization, formal analysis, methodology, project administration, validation, writing – review and editing. **Cameron Brittain:** data curation, investigation, methodology, visualization, writing – original draft.

## Funding

The authors have nothing to report.

## Consent

A signed written informed consent was obtained from the patient in accordance with the journal's patient consent policy.

## Data Availability

The data presented in this case report are available from the corresponding author upon reasonable request.
